# Cardiac output, cerebral blood flow and cognition in patients with severe aortic valve stenosis undergoing transcatheter aortic valve implantation: design and rationale of the CAPITA study

**DOI:** 10.1007/s12471-023-01826-8

**Published:** 2023-11-01

**Authors:** Astrid C. van Nieuwkerk, Kimberley I. Hemelrijk, Esther E. Bron, Anna E. Leeuwis, Charles B. L. M. Majoie, Mat J. A. P. Daemen, Justine E. F. Moonen, Alexandra de Sitter, Berto J. Bouma, Wiesje M. van der Flier, Jan Baan, Jan J. Piek, Geert Jan Biessels, Ronak Delewi, the Heart-Brain Connection Consortium

**Affiliations:** 1 Department of Cardiology, Amsterdam Cardiovascular Sciences, Amsterdam UMC, University of Amsterdam, Amsterdam, The Netherlands; 2 Biomedical Imaging Group Rotterdam, Department of Radiology & Nuclear Medicine, Erasmus MC—University Medical Center Rotterdam, Rotterdam, The Netherlands; 3 Alzheimer Center Amsterdam, Neurology, Vrije Universiteit Amsterdam, Amsterdam UMC location VUmc, Amsterdam, The Netherlands; 4 Amsterdam Neuroscience, Neurodegeneration, Amsterdam, The Netherlands; 5 Old Age Psychiatry, GGZ inGeest, Amsterdam, The Netherlands; 6 Department of Radiology and Nuclear Medicine, Amsterdam Neurosciences, Amsterdam UMC, University of Amsterdam, Amsterdam, The Netherlands; 7 Department of Pathology, Amsterdam University Medical Center, Locations AMC and VUmc, University of Amsterdam, Amsterdam, The Netherlands; 8 Epidemiology and Data Science, Vrije Universiteit Amsterdam, Amsterdam UMC location VUmc, Amsterdam, The Netherlands; 9 Department of Neurology, UMC Utrecht Brain Center, University Medical Center, Utrecht, The Netherlands

**Keywords:** Aortic valve stenosis, Transcatheter aortic valve replacement, Cerebrovascular circulation, Cognition, Neuropsychological tests, Magnetic resonance imaging

## Abstract

***Background*:**

Approximately one-third of patients with symptomatic severe aortic valve stenosis who are scheduled for transcatheter aortic valve implantation (TAVI) have some degree of cognitive impairment. TAVI may have negative cognitive effects due to periprocedural micro-emboli inducing cerebral infarction. On the contrary, TAVI may also have positive cognitive effects due to increases in cardiac output and cerebral blood flow (CBF). However, studies that systematically assess these effects are scarce. Therefore, the main aim of this study is to assess cerebral and cognitive outcomes in patients with severe aortic valve stenosis undergoing TAVI.

***Study design*:**

In the prospective CAPITA (**CA**rdiac Out**P**ut, Cerebral Blood Flow and Cognition **I**n Patients With Severe Aortic Valve Stenosis Undergoing **T**ranscatheter **A**ortic Valve Implantation) study, cerebral and cognitive outcomes are assessed in patients undergoing TAVI. One day before and 3 months after TAVI, patients will undergo echocardiography (cardiac output, valve function), brain magnetic resonance imaging (CBF, structural lesions) and extensive neuropsychological assessment. To assess longer-term effects of TAVI, patients will again undergo echocardiography and neuropsychological assessment 1 year after the procedure. The co-primary outcome measures are change in CBF (in ml/100 g per min) and change in global cognitive functioning (Z-score) between baseline and 3‑month follow-up. Secondary objectives include change in cardiac output, white matter hyperintensities and other structural brain lesions. (ClinicalTrials.gov identifier NCT05481008)

***Conclusion*:**

The CAPITA study is the first study designed to systematically assess positive and negative cerebral and cognitive outcomes after TAVI. We hypothesise that TAVI improves cardiac output, CBF and cognitive functioning.

**Supplementary Information:**

The online version of this article (10.1007/s12471-023-01826-8) contains supplementary material, which is available to authorized users.

## Introduction

Aortic valve stenosis is the most common valvular heart disease requiring intervention in Europe and the USA [1, 2]. Most cases of aortic valve stenosis are caused by calcification, leading to a rising prevalence of aortic valve stenosis in a continuously aging population [2]. Severe aortic valve stenosis causes left ventricular (LV) pressure overload and ventricular remodelling, which has a poor prognosis. If left untreated, patients will experience heart failure, functional deterioration and ultimately death [1, 2]. In addition to physical symptoms, patients with symptomatic severe aortic valve stenosis have considerably lower cognitive test scores than controls without aortic valve stenosis [3].

Transcatheter aortic valve implantation (TAVI) is a percutaneous treatment for severe aortic valve stenosis, nowadays established for low-risk patients [2]. One-third of patients scheduled for TAVI have some form of cognitive impairment prior to the intervention [3–6]. TAVI has been noted to worsen cognitive functioning but can also improve it [4–10]. Cognitive decline may be caused by cerebral emboli that can dislodge periprocedurally from the calcified aorta and aortic valve, thereby inducing brain infarctions detectable on magnetic resonance imaging (MRI) in up to 76% of patients after TAVI [5, 9]. Conversely, cognitive functioning may improve in a subset of patients after TAVI, particularly in those with baseline cognitive impairment and smaller aortic valve areas [4, 6–8, 10]. Improved cognitive functioning has been observed in multiple studies [6, 8, 10], but the underlying mechanism has not been elucidated. It has been hypothesised that restoration of impaired cardiac output after TAVI improves cerebral blood flow (CBF) and potentially also cognitive functioning [6–9].

**Fig. 1:**
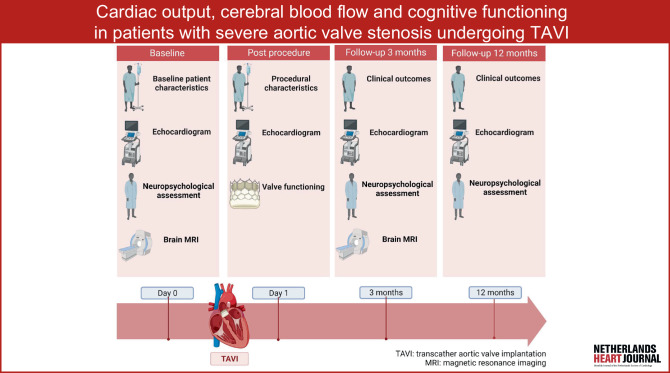
Infographic

The CAPITA (**CA**rdiac Out**P**ut, Cerebral Blood Flow and Cognition **I**n Patients With Severe Aortic Valve Stenosis Undergoing **T**ranscatheter **A**ortic Valve Implantation) study is the first larger-scale study aimed at the systematic assessment of CBF and cognition in patients undergoing TAVI (Fig. 1). We hypothesise that TAVI improves cardiac output, CBF and cognitive functioning.

## Methods

### Measurements

**Fig. 2:**
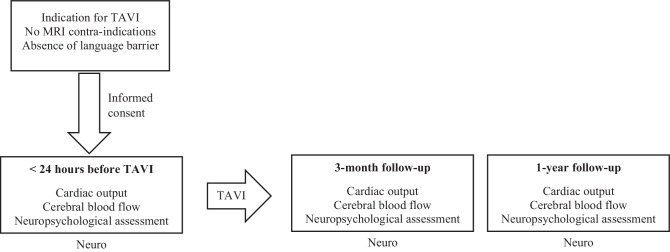
Timeline of CAPITA study. *TAVI* transcatheter aortic valve implantation, *MRI* magnetic resonance imaging

The prospective CAPITA study evaluates the effect of TAVI treatment on cardiac output, CBF, structural brain lesions and cognitive functioning. Prior to TAVI and at 3‑month follow-up, patients will undergo echocardiography (cardiac output, valve function, LV function), brain MRI (CBF using arterial spin labelling (ASL), white matter hyperintensities (WMH), structural lesions) and extensive neuropsychological testing. To evaluate longer-term haemodynamic and cognitive effects of TAVI, echocardiography and neuropsychological testing will be performed again 1 year after TAVI. Baseline measurements will be taken on the day before scheduled TAVI. Follow-up assessments will be performed during an outpatient clinic visit and are combined with a routine cardiologist consultation. Figure 2 displays the study timeline. The CAPITA study is registered at ClinicalTrials.gov (identifier NCT05481008).

### Objectives

The co-primary outcome measures are change in global CBF (in ml/100 g per min) on ASL-MRI and change in global cognitive functioning (Z-score) between baseline and 3‑month follow-up. We will also assess the relation between these co-primary outcome measures and their association with cardiac output [11].

In addition, we will explore determinants of impaired cognitive functioning prior to TAVI, e.g. demographics/risk profile, cardiac functioning and brain injury markers. Moreover, we will explore determinants of change in cognitive functioning after TAVI. As the post-procedural cognitive effects of TAVI may be transient [4, 5, 10], we will assess cognitive functioning again 1 year after TAVI.

We will assess the development and possible regression of WMH on Fluid Attenuated Inversion Recovery (FLAIR) MRI (volume and number at baseline and follow-up). WMH are a manifestation of cerebral small vessel disease, which are associated with increased risk of cognitive impairment and stroke [12]. Moreover, we will assess atrophy (brain volume on structural T_1_-weighted imaging), infarcts (structural T_1_-weighted imaging, FLAIR and diffusion-weighted imaging (DWI)) and bleeds and microbleeds (susceptibility-weighted imaging (SWI)).

### Patient population

Aortic valve stenosis is defined according to the most recent European Society for Cardiology Guidelines [2]. All consecutive patients scheduled for TAVI at the Amsterdam University Medical Centres (Amsterdam UMC) in Amsterdam, the Netherlands will be screened for study inclusion and approached for study participation at the outpatient clinic. Tab. 1 shows the inclusion and exclusion criteria for the study.

**Table 1:** Inclusion and exclusion criteria of the CAPITA study

*Inclusion criteria*	
1	Presence of severe symptomatic aortic valve stenosis and eligible for TAVI
2	Able and willing to give informed consent
3	Aged > 18 years
*Exclusion criteria*	
1	Presence of an MRI contra-indication
2	Known neurological disease, excluding cerebrovascular events without sequelae
3	Active malignant disease
4	Insufficient mastery of the Dutch language
5	Non-atherosclerotic vascular disease (e.g. vasculitis)
6	Dialysis treatment for renal failure
7	Planned surgery with general anaesthesia within 3 months after TAVI

*TAVI* transcatheter aortic valve implantation, *MRI* magnetic resonance imaging

The Amsterdam UMC is a tertiary referral hospital where approximately 350 TAVI procedures per year are performed. A multidisciplinary Heart Team decides whether a patient is eligible to undergo TAVI and selects the access route and valve type. Transfemoral access with local anaesthesia is the default approach. If transfemoral TAVI is not feasible, transaortic access is also performed. Patients will be treated with the balloon-expandable Sapien 3 or Sapien 3 Ultra (Edwards Lifesciences Inc., Irvine, CA, USA) or the self-expandable Navitor (Abbott, Abbott Park, IL, USA) or Evolut R (Medtronic Inc., Minneapolis, MA, USA) transcatheter heart valve devices.

Patients will serve as their own controls: baseline measurements in the presence of severe aortic valve stenosis are compared with post-TAVI follow-up measurement. A control group with comparable aortic valve stenosis seems to be unfeasible, as we believe it is not ethical to delay TAVI in patients with a clinical treatment indication. Time intervals for follow-up assessments are 4 weeks before or after the scheduled visit.

### Cardiac output

Cardiac output will be assessed by echocardiographic Doppler measurements of the LV outflow tract velocity time integral, which has been validated against thermodilution, pulmonary artery catheter measurements and cardiac MRI [13–15]. Transthoracic echocardiography (GE Medical Systems, Horten, Norway) will be performed by trained echocardiographists from the Amsterdam UMC according to the procedural guidelines of the European Association of Cardiovascular Imaging [16]. All echocardiographs will be evaluated by an independent core laboratory blinded for outcomes using an automatic segmentation method and visually checked by the same physician, who is blinded to clinical data and outcomes.

### Brain magnetic resonance imaging

Brain MRI will be performed using the same 3T MRI scanner (Ingenia, Philips, Best, the Netherlands) at baseline and 3‑month follow-up. Tab. 2 shows the sequences included in the MRI protocol (structural T_1_-weighted imaging, FLAIR, ASL, SWI and DWI).

**Table 2:** Magnetic resonance imaging study protocol

Scanning sequence	Objective
T_1_-weighted	Brain volume, segmentation purposes
Fluid attenuation inversion recovery T_2_-weighted	White matter hyperintensities
Arterial spin labelling	Cerebral blood flow (labelled image)
M0^a^	Cerebral blood flow (control image)
Q flow	Carotid artery flow (in ml/min)
Susceptibility-weighted imaging	Bleeds and microbleeds
Diffusion-weighted imaging	Recent and semi-recent infarctions

^a^ M0 is control image of cerebral blood flow measurement

**Fig. 3:**
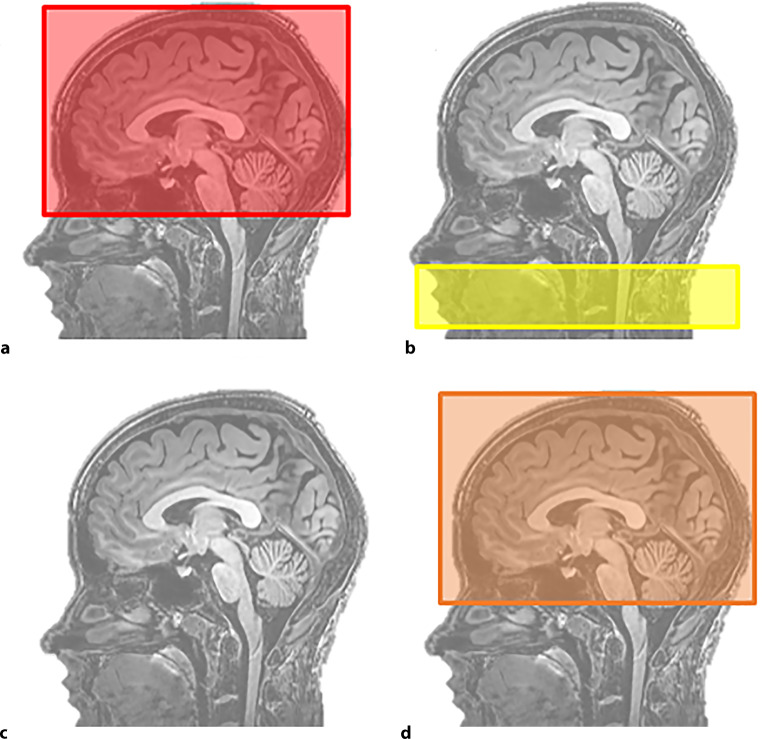
Arterial spin labelling magnetic resonance imaging acquisition. **a** Unlabelled scan, **b** endogenous tracer labelling in carotid artery, **c** labelled blood enters cerebral circulation and **d** labelled scan. Cerebral blood flow = labelled scan—unlabelled scan

ASL-MRI assesses global and regional CBF (in ml/100 g per min) using an endogenous tracer [17]. The blood will be magnetically labelled in the carotid artery, creating a labelled image and control image. Both sequences include measurements of the static brain, and the difference between these sequences is the magnetisation of inflowing blood. The ASL-MRI acquisition is shown in Fig. 3. Time of day during scanning, room temperature and visuo-auditory stimuli will be kept similar during baseline and follow-up visits. Patients will be instructed to refrain from caffeine for 3 h and from alcohol and smoking for 12 h prior to the scan [18]. ASL-MRI analyses will be performed by an independent core laboratory using pipelines, which are blinded to clinical data and whether scans were performed at baseline or follow-up. Analyses are corrected for brain volume and partial volume effects.

WMH are quantified using the Lesion Segmentation Toolbox, an automated segmentation method that uses structural T_1_-weighted imaging and FLAIR sequences [19]. With these segmentations, we will compute WMH location and volumes (in ml). More details of the MRI protocol can be found in Table S1 in the Electronic Supplementary Material.

### Neuropsychological assessment

For cognitive screening, we will use the Mini-Mental State Examination (MMSE) and Montreal Cognitive Assessment (MoCA) [20, 21] MMSE is more sensitive for detection of dementia, whereas MoCA was specifically designed to capture milder forms of vascular cognitive impairment. All patients undergo an extensive and standardised neuropsychological assessment [22, 23]. This test battery covers global cognitive functioning and 4 major cognitive domains: memory, executive functioning, attention/psychomotor speed and language [24]. Tab. 3 presents an overview of the cognitive tests and domains.

**Table 3:** Neuropsychological test battery^a^

Cognitive domain	Cognitive tests
Memory	15-Word-Auditory Verbal Learning Test: total immediate recall, delayed recall and recognition score Visual Association Test: part A
Language	Visual Association Test: naming
Attention and psychomotor speed	Trail Making Test: part A Stroop Colour Word Test: card I and II Letter Digit Substitution Test Digit Span: forward condition
Executive functioning	Trail Making Test: index score of part B/A Stroop Colour Word Test: interference score Digit Span: backward condition
Global cognition	Mean score of 4 domains
Global cognitive screening	Montreal Cognitive Assessment Mini-Mental State Examination Modified Telephone Interview for Cognitive Status [25]^b^ Telephone Montreal Cognitive Assessment [25]^b^
Depressive symptoms	Geriatric Depression Scale
Apathy symptoms	Starkstein Apathy Scale
EuroQol-5D including Visual Analog Scale	Health-related quality of life

^a^ For references to individual tests, see Table S2 in Electronic Supplementary Material

^b^ If neuropsychological assessment cannot be performed in person, Modified Modified Telephone Interview for Cognitive Status and Telephone Montreal Cognitive Assessment will be used, which can be conducted via telephone

Neuropsychological tests will be performed by trained clinical neuropsychologists blinded to previous cognitive scores and imaging findings. Time of day, location and test sequence will be kept similar during baseline and follow-up visits. At 3 months and 1 year after TAVI, the neuropsychological assessment will be repeated. If patients are unable or unwilling to visit the Amsterdam UMC during follow-up, a telephone neuropsychological assessment will be offered, including the Modified Telephone Interview for Cognitive Status and the Telephone MoCA [25]. All neuropsychological assessments include the Geriatric Depression Scale (depressive symptoms), Starkstein Apathy Scale (symptoms of apathy) and EuroQol-5D including Visual Analog Scale [22, 23].

Neuropsychological test scores are standardised into Z‑scores, which are constructed as: (test score − mean baseline score) / standard deviation of test score. Z‑scores from available tests of each cognitive domain are averaged to create 4 cognitive domain scores. Patients will serve as their own controls, and Z‑scores are based on baseline cognitive test scores for baseline and follow-up. Detailed information on the neuropsychological assessment and analyses are presented in Tables S1 and S2 in the Electronic Supplementary Material.

### Other study variables

Other outcomes and adverse events will be defined and reported according to the third Valve Academic Research Consortium and NeuroARC consensus guidelines [24, 26].

### Sample size

Based on our pilot data [11], we hypothesise a 10% increase in CBF after TAVI. With an 80% power and α of 0.05, the required sample size is 126. If a TAVI procedure is complicated by the need for permanent pacemaker implantation, subsequent MRI follow-up will be contra-indicated. In addition, some patients will not be able to take part in all follow-up measurements due to concomitant disease, institutionalisation or death. As we estimate 21% of the patients will not be able to undergo follow-up MRI, a total of 152 patients are needed.

### Statistical analysis

Clinical data will be entered at CastorEDC.com to create the final dataset. Continuous variables will be tested for normality distribution with visual inspection and the Shapiro-Wilk test. Accordingly, differences between baseline and 3‑month follow-up in CBF, as well as cognitive global and domain-specific Z‑scores, will be tested with the paired-samples *t*-test or Wilcoxon signed-rank test.

Change in global CBF (ml/100 g per min increase from baseline) will be assessed as a predictor of change in cognitive functioning (Z-score) using mixed models analysis adjusted for sex, age and education. Additionally, change in cardiac output (in l/min) will be assessed as a predictor of change in CBF with mixed models analysis. Other clinical parameters, including blood pressure, will be assessed as potential confounders of change in CBF and cognitive functioning. Finally, we will examine the association of 1‑year cognitive Z with CBF and cognitive functioning during prior visits.

### Ethical consideration

The study is conducted in accordance with the Declaration of Helsinki and the Dutch Medical Research Involving Human Subjects Act (*Wet medisch-wetenschappelijk onderzoek met mensen*). The study protocol was approved by the Medical Ethics Committee of the Amsterdam UMC. All patients must provide written informed consent before participating.

### Current status and timeline

The first patient was included in August 2020, and the last patient’s baseline visit was performed in October 2022. One-year follow-up visits are scheduled and ongoing.

## Discussion

The CAPITA study is the first study that will extensively assess cognitive and cerebral outcomes in patients with severe aortic valve stenosis undergoing TAVI. In this population, cognitive impairment is prevalent in 21–39% of patients [3, 4, 6, 8]. The pathophysiology of this impairment is most likely multifactorial: increased WMH prevalence at baseline, restricted cardiac output, and overlapping risk factors for cognitive impairment and aortic valve stenosis [1, 3, 11]. Treating aortic valve stenosis with TAVI may improve cardiac output [11] and potentially CBF.

CBF remains relatively constant within an autoregulatory range due to wall changes in cerebral vessels. Autoregulatory mechanisms of CBF are sensitive to changes in CO_2_ levels, mean arterial pressure and neurovascular signalling [27, 28]. Aortic valve stenosis may limit cardiac output to such an extent that it impairs CBF. Restoration of restricted cardiac output following TAVI may shift the autoregulatory curves to a new steady state, thereby increasing CBF [27]. In a pilot study, we found that every litre increase in cardiac output was associated with an 8.2% increase in global CBF [11].

Other studies showed that cognitive functioning is improved after TAVI in 11–19% of patients [4, 6–10]. Cognitive improvement was particularly seen in patients with pre-existing cognitive impairment and in those with smaller pre-interventional aortic valve areas [6–8]. In the general population, impaired CBF is associated with future cognitive decline and incident dementia [29]. If TAVI indeed improves CBF, it may protect against future cognitive decline. In patients with heart failure, the relation between reduced CBF and cognition has been more extensively studied. Here, heart failure treatment was associated with restored CBF and improved cognition, as observed after captopril initiation, cardiac resynchronisation therapy and heart transplantation [27]. Impaired CBF in heart failure patients correlates with disease severity and is predictive of clinical deterioration and death [30]. Hence, in patients with severe aortic valve stenosis, CBF may ultimately be used as an additional parameter for evaluation of disease severity.

The systematic approach of the current study also enables identification of potential risk factors for adverse cerebral and cognitive outcomes in patients undergoing TAVI. Pre-existing vascular risk factors for cognitive impairment and WMH prevalence may not improve after TAVI. Moreover, there is ample evidence of new brain lesions on MRI in the majority of patients undergoing TAVI [5, 9]. Therefore, the CAPITA study will also assess MRI measures of structural brain damage, i.e. microbleeds, infarcts and WMH at both baseline and 3 months after TAVI.

Personalised care and patient selection can be optimised if we can predict in which patients cognitive functioning improves after TAVI. Additionally, improvement of CBF and cognitive functioning following TAVI may suggest that cardiac interventions can benefit the brain.

### Study limitations

As the study was started during the COVID-19 pandemic, more attrition bias may be expected. However, we implemented telephone follow-up visits for patients who are unable to visit the hospital. In addition, baseline measurements can be impacted by distress due to the TAVI procedure, potentially affecting the neuropsychological assessment and outcomes. However, neuropsychologists are aware of the upcoming hospitalisation and practice empathy and compassion towards all patients, potentially minimising the confounding effects of stress.

## Conclusion

The CAPITA study is the first study to prospectively and systematically assess both positive and negative cerebral and cognitive outcomes in patients with symptomatic severe aortic valve stenosis undergoing TAVI. We hypothesise that TAVI improves cardiac output, CBF and cognitive functioning. The CAPITA study may facilitate risk stratification for adverse cerebral outcomes. CBF may ultimately be an indicator of severity of aortic valve stenosis. Furthermore, the study may render proof of concept that cardiac interventions can benefit the brain.

### Supplementary Information

**Table S1** Supplementary methods

**Table S2** Construction of cognitive domains by test scores
