# Supplementary material for: Cardiac output, cerebral blood flow and cognition in patients with severe aortic valve stenosis undergoing transcatheter aortic valve implantation: design and rationale of the CAPITA study
Source: Neth Heart J. 2023 Nov 1;31(12):461–70. doi: 10.1007/s12471-023-01826-8 (PMC10667193; doi:10.1007/s12471-023-01826-8)
Supplement: Supplementary file 1 — Table S1 Supplementary methods [file 12471_2023_1826_MOESM1_ESM.docx]

**Table S1** Supplementary methods

**ASL-MRI**

The MRI-scanner is equipped with a 16-channel DStream Head-Spine coil and foam padding to restrict head motion. A documentary about the natural world is shown during scanning and was kept similar during all baseline and follow-up scanning. Instructions were given at the same time points in the scanning protocol. The ASL measurement is performed when patients are already lying 15 minutes flat in the scanner. An experienced neuroradiologist reviews the scans visually for incidental findings and quality control.

The pseudo-continuous ASL sequence was performed with a gradient-echo single-shot echo-planar imaging readout with the following parameters: matrix size = 80x80 voxels, voxel-size = 3.0 x 3.0 mm, 19 axial slices with 7.0 mm thickness without a slice-gap, echo time/repetition time = 17/4445 ms, SENSE = 2.5, initial post-label delay = 1800 ms; slice readout time = 35 ms; resulting post-label delay range for 19 slices = 1800-2465 ms, labelling duration = 1800 ms. Fourty control-label pairs were acquired for each scan with a total scan duration of 4:44 minutes. The labelling plane was positioned 90 mm inferior and parallel to the center of the imaging volume (the anterior-commissure - posterior-commissure line). A 1x1x1 mm 3D T1-weighted scan was included in the scanning protocol for segmentation and registration purposes.

We apply a region-of-interest segmentation method and arterial spin labelling (ASL) quantification method (1-3). The region of interest is segmented using grey matter and white matter segmentation and multi-atlas registration: 30 atlases with 83 structural brain regions based on T1-weighted scans (4,5,6). Raw ASL data quantification into cerebral blood flow includes partial volume correction and motion correction (7,8). This pipeline results in average cerebral blood flow (mL/100g/min) globally and per region of interest.

**Neuropsychological assessment**

Cognitive functioning was assessed using a standardized neuropsychological test protocol. All tests were performed in Dutch. Prior to the neuropsychological assessment, two tests for cognitive screening are performed: Montreal Cognitive Assessment (MoCA) and Mini-Mental State Examination (MMSE) (9,10). Z-scores are constructed for each test according to the following formula: (test score – mean baseline score)/ standard deviation of baseline score. Z-scores for follow-up measurements are created with using baseline scores: (follow-up test score – mean baseline score)/ standard deviation of baseline score. Higher test scores indicate better performance. If higher raw tests scores indicated worse performance, z-scores are inverted for trail making test (11), 15-word-auditory verbal learning test: recognition (12), and Stroop color word tests (13).
Four cognitive domains are created: memory, language, attention and psychomotor speed, and executive functioning. A score for global cognitive functioning was created as mean z-score from the four domains. Cognitive domains are constructed by mean Z-scores of cognitive test scores. If at least one test was performed, a mean z-score is created. The interference score of Stroop color word test is calculated as card III/([card I + card II] / 2). Z-scores at baseline and follow-up are compared using paired t-tests.

**Supplementary references**

1. Aggarwal SK, Delahunty Rn N, Menezes LJ, Perry R, Wong B, Reinthaler M, et al. Patterns of solid particle embolization during transcatheter aortic valve implantation and correlation with aortic valve calcification. Journal of interventional cardiology. 2018;31(5):648-54.
2. Bron EE, Steketee RM, Houston GC, Oliver RA, Achterberg HC, Loog M, et al. Diagnostic classification of arterial spin labeling and structural MRI in presenile early stage dementia. Hum Brain Mapp. 2014;35(9):4916-31.
3. Schrantee A, Tamminga HG, Bouziane C, Bottelier MA, Bron EE, Mutsaerts HJ, et al. Age Dependent Effects of Methylphenidate on the Human Dopaminergic System in Young vs Adult Patients With Attention-Deficit/Hyperactivity Disorder: A Randomized Clinical Trial. JAMA Psychiatry. 2016;73(9):955-62.
4. Ashburner J, Friston KJ. Unified segmentation. Neuroimage. 2005;26(3):839-51.
5. Gousias IS, Rueckert D, Heckemann RA, Dyet LE, Boardman JP, Edwards AD, et al. Automatic segmentation of brain MRIs of 2-year-olds into 83 regions of interest. Neuroimage. 2008;40(2):672- 84.
6. Hammers A, Allom R, Koepp MJ, Free SL, Myers R, Lemieux L, et al. Three-dimensional maximum probability atlas of the human brain, with particular reference to the temporal lobe. Hum Brain Mapp. 2003;19(4):224-47.
7. Huizinga W, Poot DH, Guyader JM, Klaassen R, Coolen BF, van Kranenburg M, et al. PCAbased groupwise image registration for quantitative MRI. Med Image Anal. 2016;29:65-78.
8. Asllani I, Borogovac A, Brown TR. Regression algorithm correcting for partial volume effects in arterial spin labeling MRI. Magn Reson Med. 2008;60(6):1362-71.
9. Nasreddine ZS, Phillips NA, Bédirian V, Charbonneau S, Whitehead V, Collin I, Cummings JL, Chertkow H. The Montreal Cognitive Assessment, MoCA: a brief screening tool for mild cognitive impairment. J Am Geriatr Soc. 2005 Apr;53(4):695-9.
10. Folstein MF, Folstein SE, McHugh PR. "Mini-mental state". A practical method for grading the cognitive state of patients for the clinician. J Psychiatr Res. 1975 Nov;12(3):189-98.
11. Reitan RM. Validity of the Trail Making Test as an indicator of organic brain damage. Perceptual and motor skills. 1958 8.3:271-276.
12. Saan R, Deelman B. De 15‐woordentest A en B (een voorlopige handleiding). Groningen, The Netherlands: AZG: Afdeling Neuropsychologie; 1986.
13. Van der Elst W, Van Boxtel MP, Van Breukelen GJ, Jolles J. The Stroop color-word test: influence of age, sex, and education; and normative data for a large sample across the adult age range. Assessment. 2006 Mar;13(1):62-79.
