# Supplementary material for: Cardiac output, cerebral blood flow and cognition in patients with severe aortic valve stenosis undergoing transcatheter aortic valve implantation: design and rationale of the CAPITA study
Source: Neth Heart J. 2023 Nov 1;31(12):461–70. doi: 10.1007/s12471-023-01826-8 (PMC10667193; doi:10.1007/s12471-023-01826-8)
Supplement: Supplementary file 2 — Table S2 Construction of cognitive domains by test scores [file 12471_2023_1826_MOESM2_ESM.docx]

**Table S2** Construction of cognitive domains by test scores

| **Cognitive domain** | **Cognitive tests** |
| --- | --- |
| Memory | 15-word-auditory verbal learning test: total immediate recall, delayed recall, and recognition score (12) Visual association test: part A (14) |
| Language | Visual association test: naming (14) |
| Attention and psychomotor speed | Trail making test: part A (11) Stroop color word test: card I and II (13) Letter Digit Substitution Test (15) Digit Span: forward condition (16) |
| Executive functioning | Trail making test: index score of part B/A (11) Stroop color word test: interference score (13) Digit Span: backward condition (16) |
| Global cognition | Mean score of 4 domains |
| Global cognitive screening | Montreal Cognitive Assessment (17)  Mini-Mental State Examination (18)  Modified Telephone Interview for Cognitive Status** (19)  Telephone Montreal Cognitive Assessment**(20) |
| Depressive symptoms | Geriatric Depression Scale (21) |
| Apathy symptoms | Starkstein Apathy Scale (22) |
| EuroQol-5D incl Visual Analog Scale | Health-related quality of life (23) |

** Used for telephone follow-up

**Supplementary references**

11. Reitan RM. Validity of the Trail Making Test as an indicator of organic brain damage. Perceptual and motor skills. 1958 8.3:271-276.
12. Saan R, Deelman B. De 15‐woordentest A en B (een voorlopige handleiding). Groningen, The Netherlands: AZG: Afdeling Neuropsychologie; 1986.
13. Van der Elst W, Van Boxtel MP, Van Breukelen GJ, Jolles J. The Stroop color-word test: influence of age, sex, and education; and normative data for a large sample across the adult age range. Assessment. 2006 Mar;13(1):62-79.
14. Lindeboom J, Schmand B, Tulner L, Walstra G, Jonker C. Visual association test to detect early dementia of the Alzheimer type. J Neurol Neurosurg Psychiatry. 2002 Aug;73(2):126-33.
15. van der Elst W, van Boxtel MP, van Breukelen GJ, Jolles J. The Letter Digit Substitution Test: normative data for 1,858 healthy participants aged 24-81 from the Maastricht Aging Study (MAAS): influence of age, education, and sex. J Clin Exp Neuropsychol. 2006 Aug;28(6):998-1009.
16. Lindeboom J, Matto D. Cijferreeksen en Knox blokken als concentratietests voor ouderen [Digit series and Knox cubes as concentration tests for elderly subjects]. Tijdschr Gerontol Geriatr. 1994 May;25(2):63-8.
17. Nasreddine ZS, Phillips NA, Bédirian V, Charbonneau S, Whitehead V, Collin I, Cummings JL, Chertkow H. The Montreal Cognitive Assessment, MoCA: a brief screening tool for mild cognitive impairment. J Am Geriatr Soc. 2005 Apr;53(4):695-9.
18. Folstein MF, Folstein SE, McHugh PR. "Mini-mental state". A practical method for grading the cognitive state of patients for the clinician. J Psychiatr Res. 1975 Nov;12(3):189-98.
19. Katz MJ, Wang C, Nester CO, Derby CA, Zimmerman ME, Lipton RB, Sliwinski MJ, Rabin LA. T-MoCA: A valid phone screen for cognitive impairment in diverse community samples. Alzheimers Dement (Amst). 2021 Feb 5;13(1):e12144.
20. Hlávka JP, Yu JC, Lakdawalla DN. Crosswalk between the Mini-Mental State Examination and the Telephone Interview for Cognitive Status (TICS-27/30/40). Alzheimers Dement. 2022 Nov;18(11):2036-2041.
21. Almeida OP, Almeida SA. Short versions of the geriatric depression scale: a study of their validity for the diagnosis of a major depressive episode according to ICD-10 and DSM-IV. Int J Geriatr Psychiatry. 1999 Oct;14(10):858-65.
22. Starkstein SE, Mayberg HS, Preziosi TJ, Andrezejewski P, Leiguarda R, Robinson RG. Reliability, validity, and clinical correlates of apathy in Parkinson's disease. J Neuropsychiatry Clin Neurosci. 1992 Spring;4(2):134-9.
23. The EuroQol Group. EuroQol*—a newfacility for the measurement of health-related quality of life. Health Policy (New York) 1990; 16:199–206.
